# Editorial: Mechanisms and Effectiveness of Complementary and Alternative Medicine for Pain Management

**DOI:** 10.3389/fpain.2022.863751

**Published:** 2022-02-23

**Authors:** Nobuhiro Watanabe, Mathieu Piché

**Affiliations:** ^1^Department of Autonomic Neuroscience, Tokyo Metropolitan Institute of Gerontology, Tokyo, Japan; ^2^Departement of Anatomy, Université du Québec à Trois-Rivières, Trois-Rivières, QC, Canada

**Keywords:** chronic pain, complementary and alternative medicine, chiropractic, hypnosis, pain management, spinal manipulation, brain, spinal cord

Chronic pain is a debilitating condition with dramatic personal, economic and societal impacts. It affects ~20 % of the population worldwide (1) and its clinical management remains challenging. In this regard, complementary and alternative therapies are used to relieve pain symptoms together with conventional medicine (complementary) or in place of conventional medicine (alternative). Complementary and alternative medicine comprises several approaches, including but not limited to natural products (e.g., herbs, probiotics, dietary supplements), physical and manipulative interventions (e.g., physical therapy, chiropractic, osteopathy) and mind and body practices (e.g., acupuncture, yoga, meditation, hypnosis). With the growing number of patients using complementary and alternative medicine (2), research is needed to determine the mechanisms of these interventions and which are most effective for which pain conditions. This Research Topic includes seven articles on chiropractic spinal manipulation and hypnosis.

During acute pain and the transition from acute to chronic pain, pathological processes occur in the peripheral and central nervous systems. These processes lead to primary and secondary hyperalgesia. Two articles in this Research Topic examined the effect of spinal manipulation on experimentally induced hyperalgesia. Provencher et al. used a capsaicin model to examine the effect of spinal manipulation on primary heat hyperalgesia evoked by laser stimulation. Laser stimulation on the skin area sensitized by capsaicin (T_9_) enhanced pain sensation and laser-evoked cortical responses. However, pain and cortical responses were unaffected by segmental spinal manipulation (applied to the T_7−8_ joints). This result suggests that segmental spinal manipulation may not reduce primary hyperalgesia or that the transient inhibitory effect is occluded by the facilitation produced by the mechanical stimulation of sensitized tissues caused by spinal manipulation itself. Gevers-Montoro, Provencher, Northon, et al. investigated the effect of spinal manipulation on secondary hyperalgesia in the same capsaicin model, by assessing pressure pain thresholds outside the capsaicin-treated area. Pressure pain thresholds were lower in capsaicin-treated participants. However, segmental but not heterosegmental spinal manipulation prevented this effect compared with the placebo intervention. Taken together, the results from these studies suggest that spinal manipulation may be more effective at reducing secondary hyperalgesia, which is caused by central sensitization, compared with primary hyperalgesia, which is mainly caused by peripheral sensitization. The results also suggest that spinal manipulative therapy may be more effective in reducing spine pain when applied to non-painful segments adjacent to the affected area, therefore preventing the stimulation of sensitized tissues.

In the study by Pasquier et al., the authors reported prognostic factors for the effects of spinal manipulation on back pain, disability, and global perceived change in patients with back pain in the thoracic region. The results indicate that treatment comfort and expectations of improvement were significantly associated with a positive response to spinal manipulation. These results highlight the importance of considering psychological factors for research on spinal manipulation and clinical practice.

In addition to these original research articles, three review articles on chiropractic and related themes were included in this Research Topic. Gevers-Montoro, Provencher, Descarreaux, et al. reviewed clinical studies that examined the effects of spinal manipulation on neck and back pain. The results indicate that spinal manipulation is as effective as commonly used therapies such as standard medical care (analgesic medication and home exercise programs) and physical therapy. Accordingly, spinal manipulation is now recommended in combination with exercise for neck pain. However, the effects of spinal manipulation are unclear when compared to a placebo intervention or no intervention. Thus, future high-quality studies that include a well-designed placebo intervention are needed. Mercier et al. reviewed studies on devices that measure the force-time characteristics of spinal manipulation and mobilization. The factors favorable to the use of a device are user-friendliness, high versatility, high measurement performance, cost, durability, applicability to manipulative procedures, and information feedback. Such devices are also used as tools for assessing spine biomechanics, training procedures, and teaching. Therefore, the authors expect that the results of this review will help in device selection based on needs and applications. Harman et al. presented a systematic review on clumsiness associated with neck pain and injury. Seventeen of the 18 included studies reported a deterioration in performing upper limb kinesthetic tasks when the neck was positioned toward extreme limits, even in heathy individuals. This implies that changes in sensory information from the head and neck may alter the proprioception and motor coordination of the upper limbs. Understanding the underlying neurophysiological mechanisms of this effect may contribute to the improvement of rehabilitation for patients with neck pain and clumsiness.

Lastly, Desmarteaux et al. reported the results of a fMRI study on pain regulation by hypnosis. The original question addressed in the study was how verbal suggestions of hyperalgesia and hypoalgesia provided during hypnosis are transformed into predictive signals to regulate pain perception. Compared with the control condition, changes in activity in the parietal operculum, anterior midcingulate cortex and left parahippocampal gyrus during verbal suggestions predicted (1) larger pain responses in the anterior cingulate cortex and the anterior insula, in the hyperagesia condition, and (2) smaller pain responses in the anterior cingulate cortex, anterior midcingulate cortex posterior insula and thalamus, in the hypoalgesia condition. These findings provide bases to explore the transformation of verbal suggestions into perceptual regulation for different sensory systems, which are fundamental to hypnosis neurophenomenology.

In conclusion, this Research Topic provides neurophysiological bases of and future directions for research on spinal manipulation and hypnosis, which are two interventions commonly used for pain management as complementary or alternative medicine. We hope that knowledge on the effects and applications of these and other therapies will contribute to improve interactions between different health care providers as well as the development of interdisciplinary interventions for the prevention and management of chronic pain.

## Author Contributions

NW wrote the first draft of the manuscript. MP revised the manuscript. Both authors approved the final version of the manuscript.

## Funding

The contribution of MP was supported by the Fonds de Recherche en Santé du Québec and the Fondation de Recherche en Chiropratique du Québec.

## Conflict of Interest

The authors declare that the research was conducted in the absence of any commercial or financial relationships that could be construed as a potential conflict of interest.

## Publisher's Note

All claims expressed in this article are solely those of the authors and do not necessarily represent those of their affiliated organizations, or those of the publisher, the editors and the reviewers. Any product that may be evaluated in this article, or claim that may be made by its manufacturer, is not guaranteed or endorsed by the publisher.
